# The Concurrent Validity, Test–Retest Reliability and Usability of a New Foot Temperature Monitoring System for Persons with Diabetes at High Risk of Foot Ulceration

**DOI:** 10.3390/s21113645

**Published:** 2021-05-24

**Authors:** Tim Veneman, Nicolaas C. Schaper, Sicco A. Bus

**Affiliations:** 1 Department of Rehabilitation Medicine, Amsterdam UMC, University of Amsterdam, Amsterdam Movement Sciences, Meibergdreef 9, 1105 AZ Amsterdam, The Netherlands; t.veneman@amsterdamumc.nl; 2 Department of Internal Medicine, Division of Endocrinology, Maastricht UMC, Maastricht University, P. Debyelaan 25, 6229 HX Maastricht, The Netherlands; n.schaper@mumc.nl

**Keywords:** diabetic foot, foot ulcer, home-monitoring, temperature

## Abstract

At-home foot temperature monitoring may be useful in the early recognition of imminent foot ulcers that occur through biomechanical loading in people with diabetes. We assessed the concurrent validity, test–retest reliability, and usability of a new plantar foot temperature monitoring device in 50 people with diabetes and peripheral neuropathy. We compared plantar foot temperature measurements with a platform system that consists of embedded temperature sensors with those from a handheld infrared thermometer that was used as a reference. Repeated platform assessments were compared for test–retest reliability. Usability was assessed in 15 participants who used both devices daily for two weeks at home, after which they completed a questionnaire. Agreement between devices was excellent for the metatarsal heads and heel (ICCs ≥ 0.98, LOA: −0.89 °C; 1.16 °C) and hallux and lateral midfoot (0.93 ≤ ICC ≤ 0.96, LOA: −2.87 °C; 2.2 °C), good for digits 2–5 (0.75 ≤ ICC ≤ 0.88, LOA: −5.04 °C; 2.76 °C), and poor for the medial midfoot (ICC = 0.19, LOA: −8.21 °C; −0.05 °C). Test–retest reliability was high (ICC = 0.99, LOA: −0.59 °C; 1.35 °C). Participants scored between 3.8 and 4.3 on a 5-point Likert scale for willingness to measure, ease of use, measurement comfort, and duration. In conclusion, the platform shows good concurrent validity in foot regions where most ulcers occur, good test–retest reliability, and good usability for measuring plantar foot temperature. Further research should assess the clinical validity of the platform to help prevent plantar diabetic foot ulcers.

## 1. Introduction

The lifetime risk of developing a foot ulcer in people with diabetes is as high as 19–34% and ulcer recurrence rates within one year after healing are 40% [1]. Diabetic foot ulcers are commonly caused by repetitive application of high peak pressures from being ambulatory [1,2,3]. Ulcers are the most prevalent cause of diabetes-related amputations [4,5], and have a major impact on health-related quality of life and costs for society [6]. The development of effective ulcer prevention strategies is therefore of major importance. International guidelines recommend regular foot inspection and care by professionals with frequencies dependent on the risk of ulcer development, in combination with providing patient education and therapeutic footwear that aims to accommodate the foot properly and reduce high pressures under the foot [7,8,9]. However, foot screening can be time consuming and costly for the patient when the distance travelled is far, and the interval between visits may be too long for the timely detection of an imminent foot ulcer.

To improve effectiveness in ulcer prevention, strategies for the home-environment have been developed that enable patients to self-monitor their foot status. Inflammation is an important sign of imminent foot ulceration and increases local foot temperature. The inflammation is suggested to arise from the repetitive application of relatively high peak pressures on the foot during walking. Local foot temperature measurements are easy to perform and provide an objective indication of tissue inflammation. In particular, differences in local temperature between the left and right foot can provide information before other signs have appeared [10]. Where corresponding areas on the left and right foot usually do not differ more than 1.0 °C, a temperature difference of 2.2 °C or larger is seen as abnormal [11,12,13]. In such cases, reducing ambulatory activity and/or contacting a podiatrist for further diagnosis and, if needed, offloading treatment, may normalize the temperature difference [10]. This approach, where a handheld infrared thermometer is used to daily monitor foot temperature, has been shown to be effective in preventing foot ulcer recurrence in diabetes [10,14,15,16].

Despite its efficacy, home-monitoring of plantar foot temperatures is not widely implemented. Measurements with handheld thermometers can be cumbersome for patients, involving multiple temperature recordings on both feet and the calculation of the temperature differences between corresponding locations. Furthermore, errors in placing the thermometer at the correct location on the foot are easily made and full coverage of the foot is not feasible. Technology recently developed to overcome some of these limitations include socks with embedded temperature sensors and platforms or mats including an array of temperature sensors at higher spatial resolution. Temperature socks were able to reliably and consistently collect temperature data from the wearer’s feet [17], and a temperature monitoring mat showed a high sensitivity for predicting ulceration [18]. In the Netherlands, another of those platforms has been developed, called the PodoTemp. This system comprises an array of 120 embedded temperature sensors per foot, with each sensor individually measuring the temperature on the plantar foot surface while the subject stands on the platform. Software algorithms automatically measure left-to-right temperature differences between corresponding left and right foot regions, allowing a warning signal when above-threshold temperature differences are measured. The innovative built-in algorithm enables patients to execute and analyze temperature measurements without the help of others. This is a major advantage compared to the majority of currently available foot temperature monitoring devices and can potentially be the key factor for the implementation of foot monitoring devices for use in the home environment. The platform was designed in such a way that it is a system that is easy to use and can be mass produced at relatively low costs.

For such a platform to be further tested for efficacy and implemented in preventative foot care, its concurrent validity, test–retest reliability, and ease of use have to be investigated. We therefore aimed to assess the concurrent validity, test–retest reliability, and usability of the platform system for measuring foot temperatures in people with diabetes who are at high risk of foot ulceration. We hypothesized that the platform would show good concurrent validity for all plantar foot regions, except the medial midfoot, which due to the foot arch does not make contact with the platform. Furthermore, we hypothesized that the platform would have high test–retest reliability and high usability in high-risk people. 

## 2. Materials and Methods

The study consisted of two parts. In the first part, we assessed concurrent validity and test–retest reliability of the platform system during a single visit of the participant to the medical center. In the second part, we assessed the usability of the platform system during two consecutive weeks in the participant’s home environment.

### 2.1. Subjects

For the first part of the study, we included 50 persons with diabetes mellitus who all had loss of protective sensation due to peripheral neuropathy and were classified as IWGDF diabetic foot risk category 2 or 3 or who had a foot ulcer [8]. Subject characteristics are presented in Table 1. All participants were evaluated at the multidisciplinary diabetic foot clinic of the Amsterdam UMC, location AMC. For the second part of the study, we included a random sample of 16 participants from the initial group of 50. Prior to the first testing session, participants signed the informed consent form. The Medical Ethical Committee AMC approved the study protocol and all procedures were in accordance with the Declaration of Helsinki [19].

### 2.2. Instrumentation

**Figure 1:**
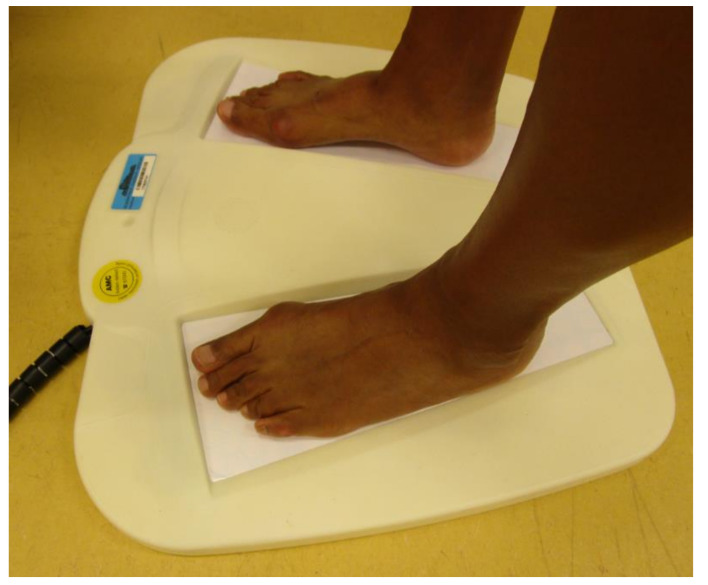
The foot temperature measurement platform system, showing the placement of the feet on the platform.

The study device was a thermometric platform that measures plantar foot temperatures (PodoTemp, PodoTemp Industries Holding BV, Eindhoven, the Netherlands) (Figure 1). It consists of two arrays of 120 temperature sensors embedded in a platform, where each individually measures the temperature at the sensor surface. The sensors have a nominal resistance value of 10 kΩ ± 1% at 25 °C, a Beta coefficient constant of 3435 K ± 1% between the temperature range of 25 °C and 85 °C, and a temperature resolution of 0.05 °C. The temperature range is specified as 20 to 45 °C. The system is designed to fit up to European foot size 46. The right and left foot are placed on the designated area with the most distal part of the toes in contact with the anterior border of the measurement area. The subject stands on the platform for 40 s to obtain equilibrium temperature readings and subsequently record the temperature in each sensor. The platform system has an innovative built-in algorithm that automatically measures left-to-right temperature differences between corresponding left and right foot regions. Temperature data were automatically saved. A prototype system was used for the study, which was connected to a laptop computer on which the temperature readings per sensor were displayed. A definitive version of the platform will incorporate warning signals when above-threshold temperature differences are recorded and will operate as a stand-alone system without the need for a laptop computer. 

As a reference condition for this study, an infrared handheld skin thermometer was used, the TempTouch^®^ (Diabetica Solutions, San Antonio, TX, USA). The TempTouch^®^ thermometer is the most commonly used equipment in studies on foot temperature monitoring and has appropriate specifications: a clinical accuracy of ± 0.5 °C within a temperature range of 18 to 41 °C. The handheld thermometer senses contact with skin, after which the device automatically triggers a temperature measurement and displays the temperature on a liquid crystal display. The thermometer incorporates a gooseneck design, which allows the user to more easily probe locations on the plantar foot.

Part 1: Concurrent validity and reliability

Demographic and medical history data were collected and a short physical examination was performed to assess loss of protective sensation, foot deformity, and IWGDF diabetic foot risk category [8]. Subsequently, subjects lay barefoot in supine position on a treatment bench for 5 min to allow for the equilibration of foot temperature. Digital photographs of the plantar surface of both feet were taken. 

Participants were then instructed to place their feet on the designated areas on the platform with their toes in contact with the anterior border and to stand still for 40 s, needed to allow for the equilibrium of foot temperature on the platform and to make a recording of the plantar foot temperature. Photographs of the feet on the platform were taken during temperature measurement to record foot placement. 

Next, the participant again lay in the supine position on the treatment bench after which the plantar foot temperatures at 14 sites under each foot were recorded using the handheld thermometer: at each toe, each metatarsal head, medial and lateral midfoot, and medial and lateral heel. Displayed foot temperatures were recorded in a clinical report form. A minimum 10 min were then taken for both the platform system and the feet of the participant to restore temperature equilibrium, after which a second temperature recording using the platform system was done. Care was taken to ensure similar foot placement as with the first platform assessment. 

**Figure 2:**
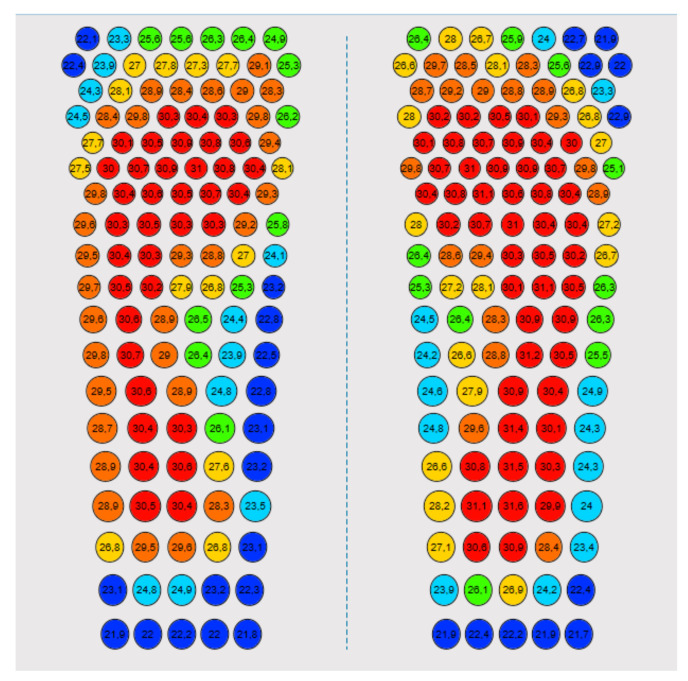
The grid of temperature sensors of the platform system with an example of temperature values recorded by each of the 120 sensors per foot. The sensor color shown in the figure indicates temperature ranges: dark blue indicates temperatures below 23.3 °C, light blue from 23.3 °C to 24.9 °C, green from 25.0 °C to 26.5 °C, yellow from 26.6 °C to 28.2 °C, orange from 28.3 °C to 29.9 °C and red above 29.9 °C.

The 14 foot regions for each foot used to assess corresponding temperatures of the platform and handheld thermometer assessments were determined using photographs of foot placement and a screenshot of temperature values per sensor during a measurement (Figure 2). 

Part 2: Usability study

Participants were equipped with a platform system and a handheld thermometer for use in their own home and were instructed to measure—on a daily basis in the morning for a period of two weeks—their foot temperature using both devices. With the handheld thermometer, participants took measurements at seven plantar locations on each foot: hallux, toe 3, 1st, 3rd, and 5th metatarsal head, midfoot, and heel. The patient recorded the elapsed time for each handheld thermometer foot assessment including the recording of temperature on a log sheet. The platform system duration of assessment for this study was set at a fixed 60 s, 40 s for data collection and 20 s for foot placement and automatic calculation of left–right foot temperature differences. After two weeks of assessments, participants completed a questionnaire pertaining to different aspects of usability: ease of use, measurement comfort, duration of measurement, and willingness to monitor regularly. The questionnaire contained open- and closed-ended questions. Close-ended questions contained a list of response options. Furthermore, statements were provided regarding user experience, which were scored on a five-point Likert scale from 1 to 5 (1 = strongly disagree, 2 = disagree, 3 = neither agree or disagree, 4 = agree 5 = strongly agree). Participants were also questioned about a preference they had for either the platform system or the handheld thermometer. 

### 2.3. Statistical Analyses

Statistical analysis was performed using SPSS statistical software (SPSS 21.0, SPSS Inc., Chicago, IL, USA). The concurrent validity of the platform system was assessed by calculating the intraclass correlation coefficients (ICCs) between temperatures measured at similar locations between the platform (first assessment) and the handheld thermometer. Left and right foot temperatures were analyzed separately. Furthermore, concurrent validity was assessed using Bland–Altman plots for assessing agreement between two methods [20]. Mean temperature differences were calculated for similar foot locations between the platform and handheld thermometer assessments for the group of 50 participants. The systematic bias between devices and the limits of agreement (LOA) were derived from the Bland–Altman plot [20].

Reliability of the platform system was assessed by calculating the test–retest agreement between repeated assessments of the platform system for 14 locations using ICCs and LOA. Concurrent validity and test–retest reliability were considered poor for ICC values less than 0.5, moderate for ICC values between 0.5 and 0.75, good for values between 0.75 and 0.9, and excellent for values greater than 0.90 [21].

For analysis of usability, closed-ended variables were summarized using frequencies, percentages, and mean Likert scores. Frequency and percentage data from the statements that were scored on a 5-point Likert scale were reduced by combining “agree” and “strongly agree” responses to form an “agree” category, and response options of “strongly disagree” and “disagree” were combined to form “disagree”. Open questions asked were assessed qualitatively. Duration of assessment with the handheld thermometer and the platform system were compared using paired t-tests. Statistical significance was set at *p* < 0.05.

As reference data on the performance of the platform or similar systems was lacking, we could not perform a power calculation but included a pre-defined convenience sample of 50 and 16 participants for the first and second parts of the study, respectively.

## 3. Results

### 3.1. Concurrent Validity

**Figure 3:**
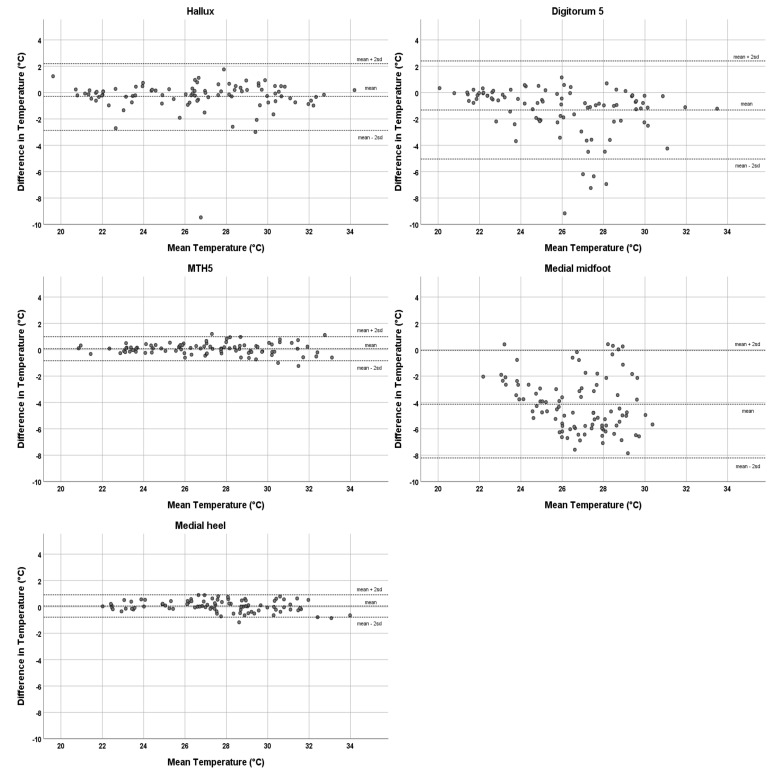
Bland–Altman plots with limits of agreement (i.e., mean ± 2sd) for measured temperature between the platform system and handheld thermometer for the hallux, digitorum 5, MTH5, medial midfoot, and medial heel. In these plots, the temperature difference between assessments (assessment platform system—assessment handheld thermometer) is shown against the average of both assessments.

The ICCs, mean differences, and limits of agreement (LOA) between platform system and handheld thermometer temperature assessments for the 14 compared foot locations are shown in Table 2. The platform showed excellent agreement with the handheld thermometer for the hallux (ICC = 0.96), metatarsal heads (ICCs = 0.99), heel (ICCs ≥ 0.98), and lateral midfoot (ICC = 0.93). Digits 2–5 showed good agreement (ICCs 0.75–0.88) and the medial midfoot poor agreement (ICC = 0.19). The mean temperature difference between devices for the parts of the foot showing excellent ICCs ranged from −0.29 °C to 0.24 °C. For digits 2–5, the mean temperature difference ranged from −1.32 °C to −0.68 °C, with the platform system showing lower temperatures; the medial midfoot showed a mean temperature difference of −4.13 °C. The upper and lower LOA were smallest in the metatarsal heads and heel, ranging between −0.89 °C and 1.16 °C. The hallux and lateral midfoot regions showed upper and lower LOA between −2.87 °C and 2.2 °C. Upper and lower LOA were −5.04 °C and 2.76 °C for the lesser digits and −8.21 °C and −0.05 °C for the medial midfoot. Bland–Altman plots for the hallux, digitorum 5, metatarsal head 5 (MTH5), medial midfoot, and medial heel are shown in Figure 3.

### 3.2. Test–Retest Reliability

**Figure 4:**
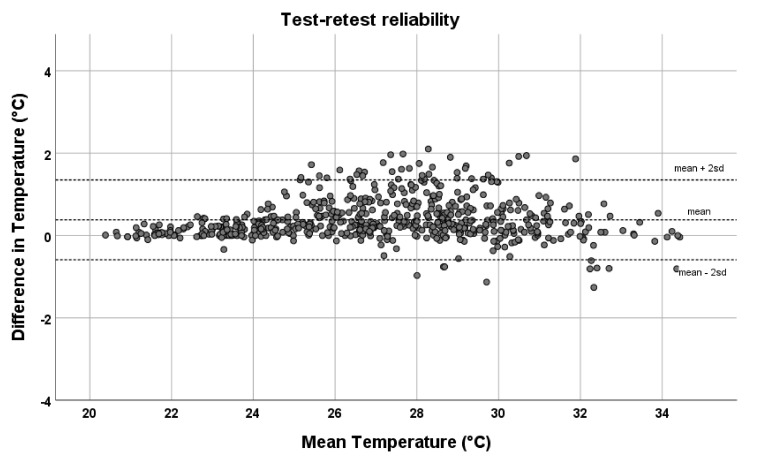
Bland–Altman plots with limits of agreement (i.e., mean ± 2sd) between repeated temperature assessments using the platform system for all 28 foot regions for both feet. In these plots, the temperature difference between repeated assessments (assessment 1–assessment 2) is shown against the average of repeated assessments.

An excellent agreement between the first and second platform system assessment was found (ICC = 0.99). Mean temperatures of assessment 1 were on average 0.38 °C higher compared to those of assessment 2. LOA values between assessments were −0.59 °C and 1.35 °C (Figure 4).

### 3.3. Usability

The Likert scores for the different statements pertaining to the user experience with using the platform system at home for a 2-week period are presented in Table 3. In general, participants agreed that the platform was easy to use (87%), and that performing a platform measurement was comfortable (80%) and quick (93%). Furthermore, ten out of fifteen (67%) participants were motivated to monitor their plantar foot temperature on a daily basis. 

No device-related adverse events were reported during the study. Furthermore, no negative or unpleasant feelings toward using the device were reported during the two-week assessment period, except for participants who had balance problems due to their peripheral neuropathy. In most cases, these balance issues could be resolved by using an external support during assessment (e.g., leaning on chair or against a wall). 

### 3.4. Assessment Time

The duration of the assessment of foot temperature was significantly lower with the platform (fixed 60.0 s) compared to the handheld thermometer (mean 133.1 s, sd 32.4), *p* < 0.01.

### 3.5. User Preference

Nine out of fifteen participants (60%) preferred using the platform to the handheld thermometer. The most important reported reason for this preference was that the platform measurement was quicker and required fewer actions from the participant in comparison to the handheld thermometer. Two participants preferred using the handheld thermometer to the platform, and four participants had no preference. 

## 4. Discussion

This study examined the concurrent validity, test–retest reliability and usability of a foot temperature monitoring platform system. The platform showed good concurrent validity for assessing plantar foot temperature of the hallux and lateral midfoot, and excellent validity for the metatarsal heads and the heel regions. The platform showed a lower and insufficient concurrent validity for assessing temperatures at the lesser toes and particularly at the medial midfoot. Outcomes showed high test–retest reliability for the platform system. Furthermore, the platform showed good usability with high scores for aspects of ease of use, measurement comfort, measurement duration, and willingness to measure. These results imply good psychometric properties for regions where plantar foot ulcers most commonly occur and, while limited to only assessing the plantar foot surface, suggest that the system can be used in clinical efficacy research.

The substantial discrepancies in concurrent validity between foot regions are caused by foot shape and the rigid surface of the platform. The plantar foot sites showing lower validity scores all made less or no contact with the measurement surface. As a result, the temperature sensors embedded in the platform were not able to accurately detect skin temperatures for these foot regions. The medial midfoot does not make contact with the measurement surface due to the medial foot arch, whereas the second to fifth toe are often raised above the ground due to the presence of claw toe deformity [22,23,24]. This is a clear limitation of the platform and rigid systems alike, although non-contact foot regions are also less likely to endure high plantar pressures and are therefore at lower risk of ulceration [25]. The hallux and lateral midfoot regions may be raised above the ground when hallux rigidus and pes cavus, respectively, is present, possibly explaining the slightly lower validity scores compared to the metatarsal heads and heel regions, which nearly always make skin contact with the platform. Most plantar foot ulcers occur at the metatarsal heads, heel, and hallux regions [26], so good to excellent criterion validity at these regions is important for clinical relevance.

The platform system showed excellent ICCs for the metatarsal head and heel regions, but compared to studies investigating other plantar foot temperature assessment tools, the LOA were comparable [27] or wider [28,29], even for the regions with excellent ICCs. This somewhat poorer performance in LOA may be explained by the characteristics for comparing regional temperatures between the platform system and the handheld thermometer as a reference. Minor inaccuracies in locating the foot regions that were compared may have been present, which for the platform were determined using photographs of foot placement and a screenshot of temperature values per sensor during a measurement (Figure 2). The plantar foot sites were not visible while standing on the platform, and therefore the sensor for comparison with the handheld thermometer recordings had to be estimated and could not be identified with absolute certainty. It is possible that specific plantar foot sites tested with the handheld thermometer were in-between two neighboring sensors of the platform. Therefore, the actual LOA of the platform may be smaller than presented in the study. Despite this study limitation, foot locations were selected with great care and the study presents a clear pattern in concurrent validity for various regions of the foot.

Test–retest reliability showed excellent agreement (ICC 0.99) between platform assessments 10 min apart. However, LOs were relatively large compared to a recent study that investigated another infrared thermometer [29]. This may be explained by the challenge in exactly repositioning the foot in the same place as with the first platform assessment. Another explanation may be related to the mean 0.38 °C higher temperature we found with the first than with the second temperature assessment with the platform system. Perhaps the feet cooled down to a larger extent in the 10 min between assessments than in the equilibrium phase of 5 min before the first assessment. This was a limitation of our measurement protocol, where we should have used equivalent time periods. After correcting for this mean baseline difference, LOA were within 1.0 °C.

Patients showed a clear willingness to measure plantar foot temperature on a daily basis using the platform system, although this was based on a period of using the device for only two weeks. The majority of participants preferred using the platform system to the handheld thermometer. Only two out of fifteen participants preferred using the handheld thermometer to the platform. Together with the high scores for ease of use, measurement comfort, and measurement duration, this suggests good usability of the platform system. 

The good concurrent validity, test–retest reliability, and usability of the platform show high potential for its use in clinical practice. However, its effectiveness in achieving the eventual goal to decrease the occurrence of diabetic foot ulcers has yet to be determined. Therefore, future research studies are needed that assess the clinical validity of the platform to help prevent plantar diabetic foot ulcers in clinical practice. This requires randomized controlled trials that evaluate the occurrence of diabetic foot ulcers over an extended period of time in individuals at high risk of foot ulceration.

## 5. Conclusions

The plantar foot temperature monitoring platform system showed good concurrent validity and test–retest reliability for plantar foot temperature assessment of the metatarsal heads, hallux, the lateral midfoot, and the heel, regions where most plantar foot ulcers occur, but showed lower validity for the lesser toes and medial midfoot. Based on a 2-week use of the platform system, participants showed their willingness to use the system on a daily basis at home, and good usability based on ease of use, measurement comfort, and duration. Further research using well-designed controlled studies is needed to assess the clinical validity of using the platform system as an at-home monitoring tool to help prevent plantar foot ulcers in persons with diabetes. 

## Figures and Tables

**Table 1:** Subject Characteristics (*n* = 50).

Characteristic	*n* or mean ± sd
Age (years) Sex (male/female) Diabetes type (1/2)	62.7 ± 8.6 40/10 9/41
BMI (kg/m^2^)	28.9 ± 5.3
History of amputations	14
Severity of deformity (no/mild/moderate/severe) ^a^	2/21/20/7
Plantar foot ulcer present (yes/no)	6/44
IWGDF risk category 2/3	29/21

^a^ Severity of deformity was classified as “no,” “mild” (i.e., presence of pes planus, pes cavus, hallux valgus, hammer toes, or lesser toe amputation), “moderate” (i.e., hallux or ray amputation, prominent metatarsal heads, or claw toes), or “severe” (i.e., Charcot deformity or [fore]foot amputation). The most severe deformity present determined classification.

**Table 2:** Agreement between platform and handheld thermometer assessments of foot temperature for 14 plantar foot regions.

	ICC	LOA		Mean diff.
		Lower	Upper	
Hallux	0.96	−2.87	2.20	−0.29
Dig. 2	0.88	−3.67	2.31	−0.68
Dig. 3	0.85	−3.89	2.37	−0.76
Dig. 4	0.77	−4.80	2.76	−1.02
Dig. 5	0.75	−5.04	2.40	−1.32
MTH 1	0.99	−0.82	1.16	0.17
MTH 2	0.99	−0.68	1.10	0.21
MTH 3	0.99	−0.66	1.08	0.21
MTH 4	0.99	−0.89	1.05	0.08
MTH 5	0.99	−0.83	0.99	0.08
Medial midfoot	0.19	−8.21	−0.05	−4.13
Lateral midfoot	0.93	−2.06	1.78	−0.14
Medial heel	0.99	−0.78	0.92	0.07
Lateral heel	0.98	−0.67	1.15	0.24

Dig, digitorum; MTH, metatarsal head. LOA: limits of agreement. LOA and mean diff. given in °C.

**Table 3:** Statements and outcomes for the usability questionnaire (*n* = 15).

Statement	Frequency (Disagree/Neither Agree or Disagree/Agree)	Mean Score
(1) The platform is easy to use	0/2/13	4.3
(2) I feel comfortable when using the platform, in relation to stepping on the platform, placement of the foot and standing on the platform for 40 s.	0/3/12	3.9
(3) The platform measurement is completed quickly	0/1/14	4.3
(4) I was motivated to measure foot temperature on a daily basis	0/5/10	3.8

Scores are given on a Likert scale, ranging from 1–5 (1 = strongly disagree, 5 = strongly agree). Frequency data were reduced by combining “agree” and “strongly agree” responses to form an “agree” category, and response options of “strongly disagree” and “disagree” were combined to form “disagree”.

## Data Availability

Not applicable.
